# Oxidative stress and redox signaling in CRPC progression: therapeutic potential of clinically-tested Nrf2-activators

**DOI:** 10.20517/cdr.2020.71

**Published:** 2021-03-19

**Authors:** Debasis Mondal, Devin Narwani, Shahnawaz Notta, Dawood Ghaffar, Nikhil Mardhekar, Syed S. A. Quadri

**Affiliations:** Debusk College of Osteopathic Medicine, Lincoln Memorial University, Knoxville, TN 37932, USA.

**Keywords:** Prostate cancer, androgen receptor, hormone therapy, endocrine resistance, oxidative stress, redox signaling, Nrf2, Nrf2-activators

## Abstract

Androgen deprivation therapy (ADT) is the mainstay regimen in patients with androgen-dependent prostate cancer (PCa). However, the selection of androgen-independent cancer cells leads to castrate resistant prostate cancer (CRPC). The aggressive phenotype of CRPC cells underscores the need to elucidate mechanisms and therapeutic strategies to suppress CRPC outgrowth. Despite ADT, the activation of androgen receptor (AR) transcription factor continues via crosstalk with parallel signaling pathways. Understanding of how these signaling cascades are initiated and amplified post-ADT is lacking. Hormone deprivation can increase oxidative stress and the resultant reactive oxygen species (ROS) may activate both AR and non-AR signaling. Moreover, ROS-induced inflammatory cytokines may further amplify these redox signaling pathways to augment AR function. However, clinical trials using ROS quenching small molecule antioxidants have not suppressed CRPC progression, suggesting that more potent and persistent suppression of redox signaling in CRPC cells will be needed. The transcription factor Nrf2 increases the expression of numerous antioxidant enzymes and downregulates the function of inflammatory transcription factors, e.g., nuclear factor kappa B. We documented that Nrf2 overexpression can suppress AR-mediated transcription in CRPC cell lines. Furthermore, two Nrf2 activating agents, sulforaphane (a phytochemical) and bardoxolone-methyl (a drug in clinical trial) suppress AR levels and sensitize CRPC cells to anti-androgens. These observations implicate the benefits of potent Nrf2-activators to suppress the lethal signaling cascades that lead to CRPC outgrowth. This review article will address the redox signaling networks that augment AR signaling during PCa progression to CRPC, and the possible utility of Nrf2-activating agents as an adjunct to ADT.

## Introduction

Despite multiple treatment strategies that are currently available against prostate cancer (PCa), it remains the second leading cause of cancer-associated deaths in elderly Caucasian men in the United States^[1]^. Furthermore, individuals of African descent show a 2-fold higher risk of developing PCa at an earlier age and manifest higher morbidity and mortality associated with advanced and metastatic disease^[2]^. Interestingly however, both the incidence of PCa and its pathologic progression are significantly lower in Orientals and other Asian populations^[3,4]^, suggesting that in addition to age and race, both epigenetics and diet may play important roles in regulating the initiation and progression of PCa^[5-8]^. Common underlying factors associated with these traits, i.e., age, race and diet, are differences in oxidative stress and antioxidant capacities in different populations, and their susceptibility to aggressive cancer growth. Indeed, clinical evidence of faster PCa disease progression in obese individuals further corroborates the hypothesis that oxidative stress and inflammation, often documented in adipose tissues, are key players in the initiation, progression and therapeutic resistance of PCa^[9,10]^. There are numerous review articles that have addressed the roles of inflammatory cytokines and reactive oxygen species (ROS) in PCa initiation and progression^[11-14]^. Therefore, this review article will focus on the significance of oxidative stress, inflammation and the redox signaling networks, which enable endocrine resistance and the outgrowth of castration resistant prostate cancer (CRPC).

Although numerous groups are currently working on this topic, a clear molecular pathway of how progression from hormone-dependent PCa cells to hormone-independent CRPC cells inevitably occurs in patients, is not thoroughly understood^[15-17]^. Indeed, the precise mechanism and the molecular steps involved in both the persistence and amplification of androgen receptor (AR) signaling, which facilitate the recurrence of PCa despite hormone deprivation, needs to be fully explored and elucidated. Since resistance to androgen deprivation plays a critical role in dictating long-term survival of PCa patients, the development of more effective treatment strategies to decrease the selection and outgrowth of CRPC cells, and especially, their lethal progression to metastatic CRPCs (mCRPC) will be of significance^[18,19]^.

We discuss the deleterious consequences of sudden hormone (androgen) deprivation in inducing oxidative stress in both PCa cells and the tumor microenvironment. Inflammatory cytokines secreted within this activated tumor microenvironment may further amplify these redox signaling networks and crosstalk with the AR signaling pathway, to enable a persistent mitogenic activation of PCa cells and the selection of CRPC^[20]^. The crucial role of interleukin-6 (IL-6), an inflammatory cytokine that is often upregulated in PCa patients and activates several inflammatory transcription factors like nuclear factor kappa B (NF-κB) and signal transducers and activators of transcription (STAT), will also be evaluated^[21]^. In addition, we will discuss the therapeutic potential of a master regulator of oxidative stress, the Nrf2-transcription factor and its antagonism of NF-κB, towards the resolution of oxidative stress and suppression of CRPC progression post hormone deprivation^[22]^. Below, we provide a short overview of PCa initiation, progression and therapeutic resistance, followed by a more thorough discussion of signaling via the AR transcription factor and its crosstalk with multiple redox signaling networks, e.g., IL-6. A short description of the oxidants and antioxidants is provided next, focused at the crucial function of Nrf2 transcription factor. Lastly, the potential of Nrf2-activating agents in suppressing CRPC outgrowth in PCa patients, will be discussed.

## Prostate cancer: initiation, progression and therapy

The prostate gland is a pseudostratified organ that provides nutrients to sperm. It also produces an alkaline prostatic fluid which helps in liquefaction of the seminal plug and neutralization of the acidic vaginal environment^[23]^. Circumstances that result in chronic oxidative stress and inflammation within prostatic microenvironments, such as aging, carcinogens, diet, obesity and genetic dysfunctions, may lead to increased cell growth (benign prostate hyperplasia or BPH) or the prostate epithelial cells may undergo oncogenic reprogramming to generate carcinomas of the prostate^[24]^. Prostate-specific antigen (PSA), an enzyme that helps liquefaction of semen, is a surrogate marker of both BPH and PCa growth, and increased serum levels of PSA is utilized for detecting early stage PCa^[25]^. However, several problems with PSA screening, especially with its inability to distinguish between BPH and PCa, has been a challenge in the clinical setting^[26]^. In addition to serum PSA levels, PCa is further confirmed by digital rectal exam and needle biopsy^[27,28]^. If confirmed at an early stage, the localized, organ confined prostate tumors can be successfully removed by prostatectomy or eliminated by radiotherapy^[24,29]^. Pathologic analysis of tumor sections from core needle biopsies can also be used to positively determine the histologic characteristics and stage of the cancer^[30]^. The Gleason grading system is commonly used to determine whether the tumor nodes are indolent, where an approach towards passive monitoring or “watchful-waiting” may be implemented. If the stained prostate cores are determined to be highly cellular, invasive and aggressive, rapid ablative therapy is necessary for therapeutic success. In this respect, a Gleason score (GS) of less than six (GS ≤ 6 or 3+3) represents slow growing and androgen responsive tumors, whereas more aggressive tumors show a GS ≥ 7 (either 3+4 or 4+3), which represent tumors having metastatic potential and a higher capacity to progress towards endocrine resistance^[29,31]^. Therefore, a precise diagnosis of indolent *vs*. aggressive PCa will decide the therapy to be implemented^[32]^.

**Figure 1:**
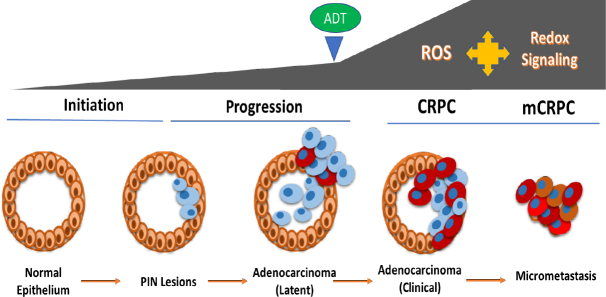
Increased oxidative stress, ROS production and redox signaling during PCa progression. A schematic representation of the prostate gland is shown on the left. Neoplastic transformation of the normal epithelium leads to PIN lesions, which progress to localized adenocarcinoma and depends on androgen and AR signaling for growth. Administration of ADT causes hormone-deprivation-induced oxidative stress, ROS production and amplified redox signaling networks in PCa cells, which facilitates the selection of CRPC cells. These aggressive CRPC cells can then metastasize to distant sites to form mCRPC, which dictates morbidity and mortality in patients. PCa: prostate cancer; ADT: androgen deprivation therapy; ROS: reactive oxygen species; CRPC: castrate resistant prostate cancer; mCRPC: metastatic CRPCs

Endocrine therapy, which uses different strategies to suppress the mitogenic action of androgens, is paramount to slow down aggressive tumor growth^[32-34]^. Androgens are steroid hormones produced by the adrenal glands and the testes, which are crucial in the mitogenic stimulation of PCa cells^[35-37]^. Therefore, ADT is the mainstay regimen in patients with aggressive or recurrent PCa^[38]^. To date, four different forms of ADT are clinically approved in patients^[39-41]^: (1) surgical removal of the testes or orchiectomy; (2) inhibitors of the CYP17 enzyme, to suppress the synthesis of androgens; (3) administration of luteinizing hormone-releasing hormone analogues/agonists to decrease adrenal steroid hormone synthesis; and (4) the use of antiandrogens, which diminish the ligand-mediated activation of AR transcription factor. However, despite the early success of ADT, cancer cells can still undergo molecular alterations and select for cells with a CRPC phenotype^[17,20,42]^. A schematic of PCa initiation, progression and therapeutic resistance is presented in Figure 1.

A rising serum PSA level, which is an AR-dependent gene product, is often an indicator of tumor recurrence in individuals following ADT. At this point, a second-generation AR antagonist, such as enzalutamide, is recommended to fully abrogate persistent AR signaling in CRPC cells^[43,44]^. However, despite its potency to suppress AR function, enzalutamide has only shown limited improvement in the long-term survival of patients^[45]^. In recent years, ADT along with low-dose chemotherapy, e.g., docetaxel, has been prescribed to patients in whom prostatectomy or radiotherapy have failed^[46]^. Interestingly, several recent studies have also shown that, in lieu of endocrine therapy, initiation with late-stage chemotherapy, e.g., docetaxel or cabazitaxel, in combination with prednisone^[47]^ or a combination of immunotherapy (sipuleucel T) and radiotherapy (Radium-223) can be used to suppress progression to high grade tumors and to decrease the possibility of endocrine resistance development^[48,49]^. These observations suggest that current hormone deprivation strategies for early stage PCa may need to be restructured, and their utility in CRPC patients may need to be fully rationalized.

## AR signaling, hormone deprivation and CRPC outgrowth

**Figure 2:**
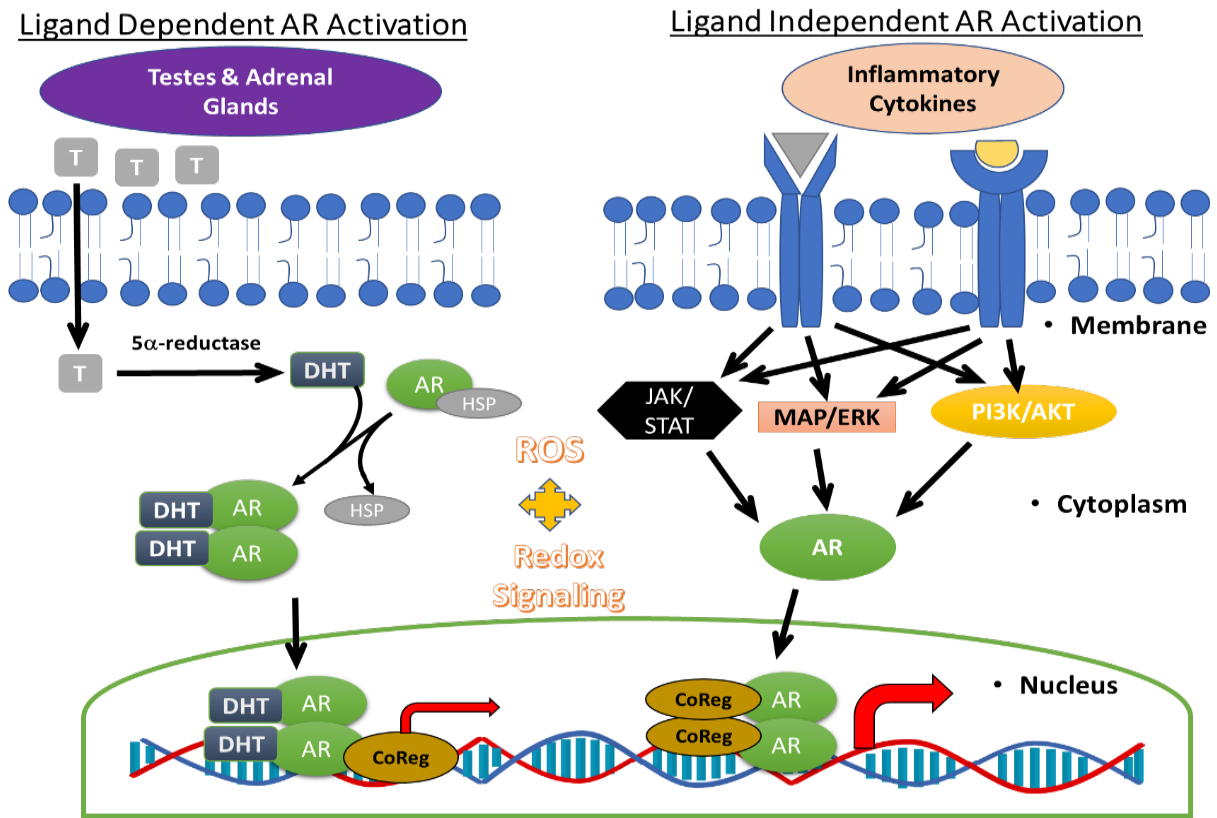
Signaling via AR occurs by both ligand-dependent and ligand-independent mechanisms. The male steroid hormones, androgen and testosterone (T) are produced by the testes and adrenal glands and manifest their endocrine effects on PCa cells via ligand-dependent activation of AR signaling (left). Inside the PCa cells, T is converted to a highly potent analog dihydrotestosterone (DHT) by the 5α-reductase enzyme. DHT binding to AR releases it from its chaperone, the heat shock proteins (Hsp). The activated AR then dimerizes and translocates to the nucleus, where it recruits coregulators of transcription to increase PCa growth. Ligand-independent activation of AR (right) can also occur due to multiple signaling pathways, primarily activated by inflammatory cytokines, and utilize ROS as second messengers. Therefore, amplified redox signaling networks can facilitate non-AR signaling, resulting in CRPC outgrowth post hormone deprivation. AR: androgen receptor; PCa: prostate cancer; ROS: reactive oxygen species; CRPC: castrate resistant prostate cancer

Decreased growth of PCa cells can be achieved by stopping the mitogenic actions of several male hormones, such as androgen, testosterone and dihydro-testosterone (DHT), the binding of which increases nuclear localization and the transactivation function of AR^[50,51]^. The AR transcription factor belongs to the family of steroid hormone receptors, and AR, a ligand (androgen) activated nuclear transcription factor that remains crucial to the growth of both PCa cells and CRPC cells^[52]^. Persistent AR signaling also seems to facilitate the metastasis of CRPC cells (mCRPC)^[53]^. Briefly, the AR protein has three major functional domains: (1) a N-terminal transactivation domain that associates with numerous transcriptional coactivators; (2) a DNA binding domain that recognizes the androgen response element (ARE); and (3) a C-terminal domain (LBD) which binds to chaperones and ligands. In androgen-dependent PCa cells, the AR protein is mostly found in the cytoplasm bound to its chaperone heat shock proteins (HSP) Hsp40 and Hsp70^[54]^. Following ligand binding, a conformational change in AR leads to its dissociation from these chaperones, resulting in its nuclear translocation. Once inside the nucleus, AR dimerizes and binds to its DNA binding element (ARE) and augments the transcription of multiple genes that are necessary for PCa survival, growth and invasion^[55-57]^. Many of the currently available antiandrogens attenuate AR signaling by competing with androgens for AR binding^[58]^ and may also be effective in suppressing the recruitment of coactivators or by activating corepressors^[59-61]^. Therefore, in androgen-dependent PCa cells, ligand-mediated activation of AR can be downregulated by multiple hormone deprivation strategies that are implemented during ADT^[62]^ and their biochemical failure enables tumor recurrence and distant metastasis^[63]^. Both canonical and non-canonical pathways of AR activation are shown below [Figure 2].

Despite the initial efficacy of ADT, the surviving cells show a persistent and often, an amplified AR signaling axis. Numerous studies have shown that an amplification of ligand-independent AR signaling occurs due to numerous crosstalk between AR and parallel non-AR signaling pathways^[17,20,42,64-66]^. Therefore, strategies to suppress both AR and non-AR signaling would be needed to ultimately suppress the outgrowth of CRPC cells. Both *in vitro* and *in vivo* studies have shown that the CRPC cells remain fundamentally dependent on AR gene expression, and their ability to sustain functional AR signaling has been linked to several different mechanisms: (1) amplification of the AR gene; (2) gain of function mutations of the AR gene; (3) intra-tumoral androgen production; (4) ligand independent activation of AR proteins; and (5) the expression of constitutively active AR splice variants^[17,20,67,68]^. The ataxia-telangiectasia-mutated (ATM) protein is a key regulator of the DNA damage response following oxidative stress. Interestingly, exposure to AR antagonists induces both telomere DNA damage and damage response inhibition in the CRPC cell line 22Rv1^[69]^. Combined exposure to enzalutamide and the ATM inhibitor, KU60019, significantly inhibits cell survival *in vitro* and tumor growth *in vivo*^[69]^. Most interestingly, our recent *in vitro* findings^[70,71]^ also showed that coexposure to potent antioxidants, such as the phytochemical sulforaphane^[70]^ and the synthetic compound, bardoxolone-methyl (CDDO-me)^[71]^ can also sensitize these 22Rv1 cells to the anti-proliferative effects of enzalutamide. Therefore, studies suggest that adjuvant therapies with potent antioxidants may be able to increase the efficacy of ADT.

## Constitutive AR signaling in PCA cells despite androgen deprivation

Multiple investigations have demonstrated the importance of AR signaling in CRPC cells, where constitutively high nuclear AR protein levels were observed in both CRPC cell lines grown in the absence of hormone stimulation^[72]^ and in both tumor samples from patients undergoing ADT^[68]^ and in circulating tumor cells (CTC) from CRPC patients^[73]^. However, little is known about the exact mechanism(s) that causes PCa cells to persistently activate a potent level of AR expression and signaling that enable increased nuclear AR levels. Frequently, it has been documented that PCa cells that manifest even a partial resistance to hormonal therapy show upregulation of factors linked with cell survival^[74]^ and downregulation of proteins associated with apoptosis^[75,76]^. Therefore, it is hypothesized that CRPC outgrowth occurs due to a significant shift in gene expression profile of the surviving PCa cells that have the potential to be more aggressive and can overcome the initial insult of hormone deprivation^[77,78]^. It has also been suggested that these initial CRPC cells can use residual levels of hormones that are secreted from adrenal and/or prostatic sources, which enable them to persistently activate AR signaling^[64,79]^. In addition, it is also evident that CRPC cells may also start expressing enzymes required for the *de novo* synthesis of testosterone and other steroid metabolizing enzymes that facilitate the conversion of androgens to the highly potent form, DHT^[80]^. Another key factor, often documented in CRPC tumors from patients, involves the expression of spliced isoforms of AR, which lack the LBD and hence, become constitutively activated^[67,81]^. In this respect, AR-V7 (also known as AR3) is recognized as a major splice variant in CRPC cells, both in solid tumor specimens and in circulating tumor cells^[82]^. Indeed, increased AR-V7 levels have often been associated with a worse prognosis in PCa patients^[25,83]^. Intriguingly, the AR-V7 protein, and its constitutive activation and nuclear levels, were also shown to support epithelial-to-mesenchymal transition of PCa cells, underscoring its importance as a predictive biomarker of aggressive disease and therapeutic resistance^[84]^. However, the precise mechanisms that control the above documented changes in AR signaling to overcome the initial effects of hormone deprivation are still not fully elucidated^[85-87]^.

Essentially, denying PCa cells of their preferred growth signaling results in a sudden change within the tumor microenvironments and induces oxidative stress. Changes in the behavior of the PCa cells undergoing ADT is likely due to an adaptive response to a combination of stresses from the hypoxic tumor environment, which results in the activation of multiple alternate second messenger signaling that increase both AR gene expression and ligand-independent AR activation^[88,89]^. Inflammatory cytokines produced within this microenvironment may further amplify second messengers via ROS and intensify redox signaling and crosstalk that enable progression to androgen independence. Indeed, recent findings show that androgen-induced expression of dynamin-related protein 1 (Drp1) can regulate mitochondrial function in PCa cells^[90]^, thus providing evidence of a vicious cycle of ROS production, inflammation and redox signaling amplification. Both *in vitro* and *in vivo* studies have demonstrated that changes in the activity of various cofactors that modify AR signaling can support its crosstalk with multiple, parallel signal transduction pathways involved in CRPC progression^[91-96]^. Indeed, endocrine resistance has often been associated with elevated levels of pro-inflammatory cytokines, such as interleukins (ILs) like IL-6 and IL-8, tumor necrosis factor-alpha (TNF-α), macrophage chemotactic factor-1 and several transforming growth factor (TGF) family of proteins like TGF-b1 and Activin. Furthermore, changes in downstream transcription factors, e.g., NF-κB, STATs and CCAAT/Enhancer binding protein-beta (C/EBP-β), which are often activated by the above mentioned inflammatory mediators, have also been implicated in augmenting AR function^[97-100]^. The invasive PCa cells may metastasize to tissue sites in which tumor hypoxia and changes in tumor vascularization may further upregulate oxidative stress and redox signaling^[101,102]^. These observations underscore the importance of signaling crosstalk in CRPC progression.

Novel strategies to suppress the outgrowth of CRPCs may be developed by using inhibitors of specific inflammatory signaling pathways or by using potent antioxidants, primarily as an adjunct to the currently available ADT regimens. An inflammatory cytokine that is of crucial importance in this respect is interleukin 6 (IL-6), which is significantly increased in aggressive tumor microenvironments^[21,91]^. Furthermore, evidence of differential IL-6 signaling in hormone-dependent and -independent PCa cells^[103,104]^ and therapeutic implications of IL-6 antagonism to suppress CRPC progression^[99,105,106]^. Therefore, we are providing below a short overview of IL-6 signaling and its role in increasing oxidative stress and redox signaling in the CRPC cells.

## Interleukin-6 in redox-signaling amplification in CRPC cells

IL-6 is a very important cytokine with respect to its potential to enhance both AR and non-AR signaling in PCa cells. It is primarily produced in response to tissue injury, inflammation, and oxidative stress^[107]^. In addition to its role in regulating immune cell function against cancer cells, it plays a crucial function in cancer survival and pathophysiology^[91,103,108]^. Androgen deprivation in mice by orchidectomy was shown to increase serum IL-6 levels and IL-6 mRNA expression in the bone marrow cells^[109]^. IL-6 is also able to promote the growth of hormone-dependent prostate cancer cells in castrated mice^[110]^. Circulating levels of IL-6 were found to be increased in patients with metastatic CRPC^[111]^ and its circulating levels correlated with poor prognosis in PCa patients^[112]^. Indeed, both IL-6 and IL6-receptor alpha (IL-6Rα) can be synthesized by PCa cells and their expression is enhanced in aggressive tumors^[113]^. Therefore, the above findings implicate IL-6 signaling as a key player in the persistent AR signaling and activation in cells with a CRPC phenotype.

**Figure 3:**
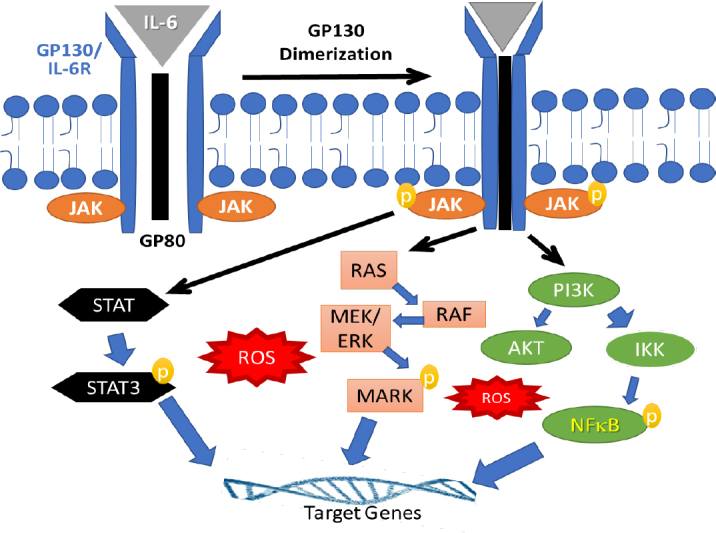
Multiple signaling cascades are activated following IL-6 binding to its receptor (GP130). Ligand binding causes GP130 dimerization and activation (phosphorylation) of the associated JAK kinase, which then activates downstream STAT transcription factor. GP130 activation can also transduce second messenger signaling via the RAS/RAF/MEK/ERK pathway to activate MAP kinase (MAPK). In addition, this inflammatory cytokine can also initiate the PI3K signaling at the membrane, which can either activate IκB kinase (IKK) and thus enhance NF-κB transcription factor function or PI3K may activate the downstream protein kinase B (AKT) which suppress NF-κB activation. Therefore, IL-6 signaling activates multiple redox signaling networks that may crosstalk with AR and activate it in a ligand-independent manner. IL-6: interleukin-6; JAK: Janus associated kinase; STAT: signal transducers and activators of transcription; NF-κB: nuclear factor kappa B

Second messenger signaling via IL-6 has been well reviewed in several past publications^[114,115]^. Briefly, IL-6 signaling is initiated by its binding to the cell membrane receptor IL-6Rα (gp80 subunit) which facilitates dimerization with the signal transducing subunit (gp130). Multiple downstream signaling cascades are activated following IL-6Rα activation, and many of these utilize ROS second messengers^[116,117]^. Following ligand binding, the receptor-associated Janus associated kinase (JAK) then activates gp130 and helps it to serve as a docking site for the STAT transcription factors. Indeed, IL-6 can simultaneously activate three distinct signal transduction pathways, which include (1) JAK/STAT signaling, especially via STAT3; (2) the mitogen-activated protein kinases/extracellular signal-regulated kinase (MAP/ERK) cascade; and the (3) phosphatidylinositol 3-kinase/protein kinase B (PI3K/AKT) cascade. These activated second messengers facilitate the stabilization, dimerization and nuclear translocation of multiple transcription factors, thus regulating many target genes^[117]^. A simplified version of the IL-6 signaling cascades is shown below in Figure 3.

Interestingly, during the early stages of carcinogenesis, high levels of circulating IL-6 may play a preventive role in tumor progression by activating basal transcription factors such as specificity protein 1 (Sp1) and CCAAT enhancer binding protein beta (C/EBP-β), also known as nuclear factor for IL-6 (NF-IL6), but during late stages of aggressive growth, IL-6 signaling may help cancer cells become more resistant to ablative therapy by inducing inflammatory transcription factors such as STAT and NF-κB^[118-120]^. Several studies have also shown that a number of transcription factors, e.g., NF-κB, STAT and C/EBP, which are activated during IL-6 signaling, can directly interact with AR and regulate its function^[121-124]^. In the aggressively growing CRPC cells, where a state of epigenetic transformation is found to be critical, activation of IL-6 and the downstream signaling to activate STAT3, PI3K and MAPK, and ultimately the nuclear translocation of NF-κB have been documented^[125]^. Altholactone, a natural compound isolated from *Goniothalamus spp*., inhibited both NF-κB and STAT3 activation and induced ROS-mediated apoptosis in PCa Cells^[126]^. The resistance of CRPC cells to antiandrogens was overcome by targeting NF-κB signaling using artesunate^[127]^. This study highlighted the combination of NF-κB inhibitors and AR antagonists may potentiate the clinical efficacy of ADT in CRPC patients. Furthermore, this combination reduced NF-κB signaling and decreased the expression of both AR and AR-V7. These studies demonstrate the importance of targeting NF-κB and IL-6 to suppress CRPC progression.

Most interestingly, although the androgen-dependent PCa cell line (LNCaP) starts dying within a few days under hormone deprived conditions (charcoal stripped serum), these cells rapidly become resistant when cultured in presence of recombinant IL-6. Similarly, the selectivity of the CRPC phenotype was also evident in LNCaP cells transfected with a constitutively active STAT3 expression vector^[128,129]^. Therefore, based on the crucial role of IL-6/JAK/STAT3/NF-κB signaling in CRPC outgrowth, one could assume that the targeting of IL-6 signaling would be an ideal approach. Although Phase-I clinical studies with a chimeric monoclonal antibody against IL-6, Siltuximab (CNTO328) combined with docetaxel, showed some promise in delaying disease progression in patients with mCRPC^[105]^, Phase-II results were not as promising^[130]^. Furthermore, studies involving monotherapy with Siltuximab have not yielded significant positive results in PCa patients^[131]^. Therefore, in accordance with current scientific evidence of the crucial role of continuous redox signaling crosstalk in CRPC cells, the use of antioxidants may provide a more promising approach. Therefore, to assist the reader’s understanding, we are providing a brief overview of pro-oxidants and antioxidants below.

## Oxidative stress, reactive oxygen species and antioxidants

Reactive oxygen species (ROS) are produced during aerobic metabolism in cells and are most often generated during the conversion of glucose into adenosine triphosphate (ATP) via the tricarboxylic acid (TCA) cycle and the mitochondrial electron transport complex (ETC)^[132]^. A dysfunctional or overwhelmed ETC can result in the leakage of electrons, which are captured by molecular oxygen [O_2_] to ultimately generate free oxygen radicals such as the superoxide anion (O_2_^-^), hydrogen peroxide (H_2_O_2_), and hydroxyl radicals (OH⋅)^[133]^. Chronic exposure to high levels of these ROS can cause significant damage to DNA, proteins and lipids, and ultimately result in decreased antioxidant defense mechanisms^[134]^. In addition to the mitochondria, another cellular organelle often involved in ROS production is the endoplasmic reticulum (ER) where proteins are translated from cellular messenger RNAs (mRNAs). A third candidate for ROS generation, at both the plasma membrane and the nuclear membrane, are NAD(P)H oxidase (NOX) enzymes, which produce ROS in response to inflammatory stimuli and act as carriers of electrons to the ETC within the mitochondria. Studies have shown that the beneficial or detrimental effects of oxidative stress are contingent upon the genetics of the cell, the kind of ROS involved, the duration and levels of ROS being generated, and the ability of cells to replenish their antioxidant capacity. Therefore, optimal regulation of oxidative stress within different parts of the cell is critical in maintaining the redox balance.

Oxidative stress results in ROS-mediated second messenger signaling, which increases the production of inflammatory cytokines and creates a feed-forward loop to generate more ROS and amplified redox-signaling^[135]^. In highly proliferative cancer cells, and especially in cells undergoing sudden changes in their microenvironments such as hormone deprivation or hypoxic conditions, the ER is susceptible to proteotoxic stress^[136]^. Under physiologic conditions, an unfolded protein response regulates protein stability via the proteasome. However, under pathologic conditions, the resultant ER-stress and altered ER-mitochondria homeostasis then cause the production of ROS and oxidative stress^[137-139]^. Communication between mitochondria and other organelles is also being documented^[139]^. Therefore, these ER-mitochondria contact sites are crucial in ROS generation, redox signaling and therapeutic resistance.

An intricate antioxidant defense system counterbalances the constant production of reactive oxidants by the aggressive prostate cancer cells^[140]^. These highly complex webs of oxidation-reduction cycles consist of donor-acceptor reactions, which ensure that the response to reactive oxidants is adequate, for both the immediate signaling needs of the cell while decreasing the long-term deleterious consequences of ROS. Therefore, ROS generated via different reactions are promptly quenched within the close vicinity of their intracellular microenvironments. Oxidative stress occurs when there is an imbalance in the regulation of ROS production and the replenishment of required antioxidant levels. In this respect, the first line of defense against ROS are the small molecule antioxidants such as glutathione (GSH), vitamin C (Ascorbic acid; AA) and vitamin E (Tocopherol) which contribute to immediate antioxidant capacity of the cell^[141]^, and hence, has been most often investigated towards anticancer therapy^[142,143]^. These small molecules scavenge ROS and prevent them from catalyzing the production of other free radicals that propagate the damage to cellular components such as DNA, protein and lipids. In addition, the peroxiredoxins (Prx) and thioredoxins (Trx) are responsible for chelating transition metals that facilitate OH* production via the Fenton and Haber-Weiss reactions^[144]^. The Trx - Trx reductase (TrxR) system also helps to maintain a reducing environment by catalyzing electron flux from nicotinamide adenine dinucleotide phosphate (NADP). Last but not the least, the regeneration of antioxidant capacities of all of these small molecules are brought about by an interacting network of redox cycles, most importantly the GSH - GSH peroxidase (Gpx) cycle that is pivotal in maintaining cellular redox homeostasis. Several past review articles have addressed the crucial role of the GSH - Gpx axis as well as the importance of the enzymes Gpx and GSH synthase in regulating tumor initiation, progression and therapeutic resistance^[145,146]^. Therefore, in addition to the small molecule antioxidants, studies are now focusing on the therapeutic potential of several of the antioxidant enzymes^[147]^. Decades of research has been done on the most crucial redox regulatory enzymes, superoxide dismutase (SOD) and catalase (CAT) which inactivate O_2_^-^ and H_2_O_2_ respectively, and their ability to rapidly quench these highly reactive oxidants. Furthermore, numerous recent studies have demonstrated the importance of numerous other antioxidant enzymes as well, such as TrxR, Gpx, heme oxygenase-1 (HO-1), Glutamate cysteine ligase catalytic (GCLc), NADPH Quinone oxidoreductase enzyme (NQO1) *etc*., the expressions of which are regulated by the master regulatory transcription factor, nuclear factor E2-related factor 2 (Nrf2)^[148-150]^. Indeed, us^[150,151]^ and others^[152,153]^ are discovering the multifaceted functions of Nrf2 in regulating tumor initiation, progression and therapeutic resistance. Since redox reactions play key roles as potent second messengers in many signaling pathways, a short list of the important oxidants and antioxidants, along with their respective enzymatic machinery and symbols, is provided in Table 1.

**Table 1:** Reactive oxidants and the ROS quenching antioxidants

Oxidants and antioxidants	Symbol^a^
A. Radical ROS	
Superoxide	O_2_^-^
Hydroxyl radical	OH
Nitric oxide	NO
Organic radical	R
Peroxyl radical	ROO
Alkoxyl radical	RO
Thiyl radical	RS
B. Non-radical ROS	
Hydrogen Peroxide	H_2_O_2_
Singlet Oxygen	O_2_
Ozone trioxygen)	O_3_
Organic hydroperoxide	ROOH
Hypochlorous acid	HOCL
Peroxynitrite	ONOO
C. Enzymatic system	
Superoxide Dismutase	SOD
Catalase	CAT
Glutathione Peroxidase	GPx
Glutathione Reductase	GR
Glutathione S-transferase	GST
Thioredoxin Peroxidase	TrxPx
Thioredoxin Peroxidase	Trxr
D. Non-enzymatic system	
Glutathione	GSH
Glutaredoxin	Grx
Thioredoxin	Trx
Peroxiredoxin	Prx
Sulfiredoxin	Srx
Vitamins A, C, E	

^a^Symbols for both oxidants and antioxidants are shown

## Is there a therapeutic benefit of antioxidants in CRPC progression?

The initial focus of oxidative stress and ROS has been mostly directed towards their deleterious effects on cellular macromolecules, e.g., DNA, protein and lipids. However, more recent studies are showing that they are often utilized as signaling molecules and play an active part in regulating numerous cellular processes^[154]^. An exquisitely regulated redox signaling is essential for various biological functions, including cell survival, cell growth, proliferation and differentiation, and immune response. Reduction and oxidation (redox) reactions play key roles as potent second messengers in cancer cells and are being targeted as novel cancer therapies^[155]^. However, it is also being increasingly realized that ROS and oxidative stress may have a dual role in cancer^[156]^. Oxidative stress can promote pro-tumorigenic signaling that increases cancer cell proliferation, survival, and adaptation to hypoxic microenvironments. On the other hand, regulated oxidative stress can also be used to promote antitumorigenic signaling and either trigger ROS-induced cancer cell apoptosis or suppress ROS-induced signaling that is essential for tumor cells. Indeed, redox reactions are able to activate signaling pathways such as mitogen activated-protein kinase (MAPK), extracellular-regulated kinase 1/2 (ERK1/2), phosphoinositide-3-kinase (PI3K), protein kinase B (AKT), and many more, which have been thoroughly reviewed by Zhang *et al*. ^[156]^ (2016). Aggressive cancer cells take advantage of these altered redox signaling networks to facilitate their proliferation and invasion capabilities, and to acquire therapeutic resistance.

Increased oxidative stress and a decreased antioxidant capacity have been previously associated with aggressive prostate cancer^[157]^. Indeed, numerous recent findings also underscore the connection between oxidative stress and AR signaling, and the risk of developing CRPC tumors^[158-160]^. Shiota *et al*.^[159]^ (2010), had documented that Twist1, a member of basic helix-loop-helix transcription factors as well as AR was upregulated in response to hydrogen peroxide, and the response to which was abolished by an addition of N-acetyl cysteine (NAC) and Twist1 knockdown. In a more recent publication, this group also showed that gene polymorphisms in antioxidant enzymes correlate with the efficacy of ADT, clearly implicating the importance of oxidative stress with CRPC outgrowth^[160]^. In this respect, ligand-receptor interactions between androgens and AR is a major regulator of redox signaling in PCa cells^[66,161-163]^ and in dictating treatment resistance^[162]^. Therefore, it is now well accepted that sudden changes in androgen levels, such as during hormone deprivation, can drastically alter cellular homeostasis to increase oxidative stress, ROS production and dysregulated redox signaling networks. Fajardo *et al*.^[163]^, (2016) showed that exposure to an oxidation product of vitamin-E, α-tocopherylquinone, decreased AR expression and protein levels. However, most interestingly, coexposure to the antioxidant form, a-tocopherol was able to abrogate this effect, suggesting that both oxidative stress and the resultant antioxidants are important in regulating AR function in androgen-responsive PCa cells. Studies have indicated that the modification of NOX expression by androgen may have significant implications on the response of PCa cells to hormone deprivation^[161,164]^ and a crucial role of both NOX4 and NOX5 has been suggested in CRPC cells by us^[151]^ and others^[165,166]^. Interestingly, a direct suppression of NOX-mediated ROS production showed significant suppression in cell proliferation and survival in PCa cells, whereas the quenching of ROS by using a GSH-mimetic was not as effective. Therefore, more potent strategies to abrogate the ADT-induced redox signaling network amplifications will be necessary to decrease CRPC selection and metastasis. Indeed, a crucial issue that is often overlooked is the role of sudden hormonal changes in facilitating the metastasis of CRPC cells, primarily to the bone marrow and brain^[167,168]^. Since androgen signaling plays an important role in normal physiology and cellular functions^[169-172]^, whole body hormone deprivation may result in systemic oxidative stress, especially in the bone marrow and brain, where the CRPC cells with an acquired redox-signaling network can find new sanctuaries and form metastatic foci, resulting in increased mortality associated with mCRPCs. Hence, potent compounds that may restore the antioxidant homeostasis in PCa patients following hormone deprivation, may not only suppress the selection of CRPC cells but may also decrease progression to metastatic CRPCs.

## NRF2 as a therapeutic target against CRPC progression

A constant high-level expression of the afore-mentioned antioxidant enzymes would be crucial to long-term protection from oxidative stress and compounds that augment their gene expression and protein levels may be of significant therapeutic value against CRPC progression. In this respect, the transcription factor nuclear factor erythroid 2-related factor 2 (Nrf2) is known to be a master regulator of oxidative stress^[173,174]^. Key findings in the field of chemoprevention has shown that the induction of Nrf2 may have significant potential in cancer. Studies in Nrf-2 knockout mice showed that the tumors generated in these mice are more aggressive and showed multiple organ metastasis^[175]^. Furthermore, the prevalence and size of colorectal cancers were increased in Nrf-2 knockout mice^[176]^. On the other hand, upregulation of Nrf-2 suppressed growth and migration of PCa cells by upregulating antioxidant proteins such as ferroportin^[177]^. However, despite the findings that oxidative stress is a potent stimulator of CRPC outgrowth (as discussed above) few studies have investigated the significance of Nrf2 signaling in CRPC cells and possible efficacy of activating the Nrf2 transcription factor to suppress CRPC progression.

In cooperation with Nrf2, a number of related proteins such as Nrf1, Bach1, and the FoxO (forkhead box, class O) family of transcription factors, especially FoxO3, are of significant importance in regulating the expression of the majority of antioxidant genes^[178,179]^. Together, these transcription factor families are responsible for ensuring that high levels of the antioxidant enzymes are produced to suppress the deleterious effects of oxidative stress and ROS. Therefore, strategies to activate these transcription factors, rather than the use of small molecule ROS quenching agents, e.g., GSH, n-acetyl cysteine (NAC), vitamin-E, vitamin-C, *etc*., may provide a more clinically applicable approach to suppress the amplified redox signaling that enables selection and progression of CRPC cells and their metastasis to sequestered sites.

Indeed, due to their potent effects, numerous recent studies are focusing on the regulation of transcriptional activity of Nrf2 in order to persistently suppress the deleterious effects of oxidative stress and the ROS-induced disease manifestations^[152,180,181]^. As explained in the previous sections, an integrated signaling network involving NF-κB and AR plays a critical role in persistent AR signaling and the development of CRPC. The transcription factor Nrf-2 is known to be a potent inhibitor of NF-κB signaling, as evidenced with numerous phytochemicals and pharmaceuticals that have shown anticancer effects^[22,182-184]^. Furthermore, our recent findings demonstrated a direct role for Nrf2 in downregulating both AR expression and function^[71,148,150-153]^.

Briefly, Nrf2 belongs to the CNC-bZIP (cap’n’collar- basic leucine zipper) family of nuclear transcription factors^[22,153,185]^. In its inactive state, Nrf2 is usually bound to its cytoplasmic chaperone, Kelch-like ECH-associated protein 1 (Keap1) which dictates Nrf2’s proteosomal degradation^[174,181]^. When cells are exposed to oxidative stress, Nrf2 dissociates from Keap1 and translocates to the nucleus. Within the nucleus, Nrf2 interacts with small Maf proteins, especially MafG, which are also bZIP-type transcription factors^[186,187]^. The Nrf2-MafG complex, along with other coactivators or corepressors, can regulate the transcription of numerous oxidative stress related genes by binding to their Electrophile Response Elements (EpRE) within promoter/enhancer regions of the DNA^[188,189]^. Nuclear localized Nrf2 and its binding to EpRE sequences can increase the expression of many antioxidant genes such as Prx-1, Txn-1, Gpx, HO-1, *etc*. Furthermore, EpRE like sequences have also been located in the promoter region of numerous other antioxidant genes including NQO1, glutathione S-transferaseA2 (GstA2), γ-glutamylcysteine synthetase (GCS), superoxide dismutase (SOD-1 and -2), ferritin and UDP-glucuronosyltransferase (UGT). Thus, a functional Nrf2 protein is essential for cell survival and several studies have shown that the knockdown of Nrf2 expression sensitizes cells to oxidative stress and apoptosis^[190,191]^. Interestingly, studies have also located several EpRE like sequences at close vicinity to the AR gene, which may regulate AR expression following oxidative stress and Nrf2 binding^[151,192]^. Findings indicate that the thioredoxin domain-containing 9, an important regulator of ROS, can activate AR signaling^[192]^. Interestingly, in human prostate cancer cells, the NF-κB inhibitor, curcumin and its potent analog ca27, were shown to down-regulate AR expression via an oxidative stress mediated mechanism^[193]^. Investigators showed that ROS production preceded AR loss and that ca27-mediated down-regulation of the AR was decreased by the antioxidant, N-acetyl cysteine.

**Figure 4:**
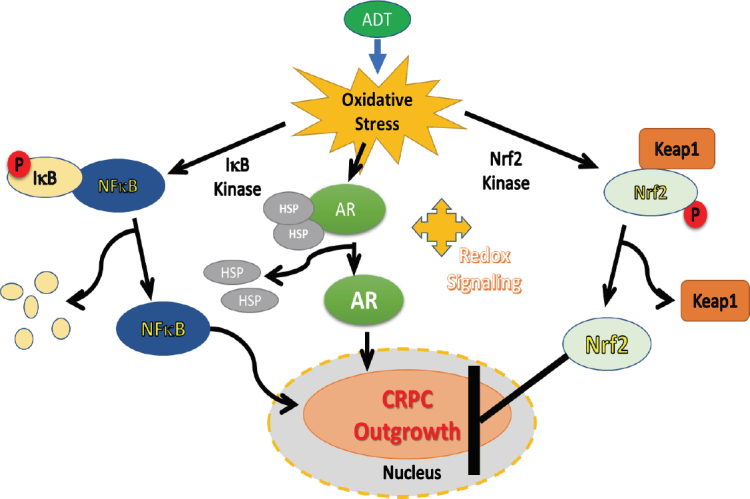
Crosstalk regulation of Nrf-2, NF-κB and AR signaling in CRPC cells. Hormone deprivation (ADT) induced oxidative stress and redox signaling activates both NF-κB and AR signaling that facilitates CRPC progression. Oxidative stress also increases Nrf2 nuclear levels which blocks both NF-κB and AR signaling. Thus, reduction of oxidative stress post-ADT by using potent Nrf2-activating agents may prevent CRPC outgrowth. CRPC: castrate resistant prostate cancer; NF-κB: nuclear factor kappa B; AR: androgen receptor

Fluctuations in hormone levels may induce inflammation within tumor microenvironments and increase both NF-κB and AR function, the extent of which can be potently regulated by nuclear Nrf2 levels. As discussed in the previous sections, following activation, both NF-κB and AR translocate into the nucleus and bind to their respective DNA response elements, leading to the expression of many genes associated with aggressive tumor growth and endocrine resistance. Oxidative stress leads to IκB kinase activation and phosphorylation of IκB, targeting it for polyubiquitination and proteasomal degradation, ultimately resulting in the release and nuclear translocation of NF-κB^[14]^. Similarly, AR is sequestered in the cytoplasm via several HSPs and translocates to the nucleus following its release due to ligand binding and dimerization^[20,52,54,79,81,98]^. The same type of mechanism of Nrf2 nuclear translocation occurs due to ROS-induced inactivation of the Keap1 protein^[173,174]^. Nuclear translocated NF-κB can directly inhibit Nrf-2 at the transcriptional level^[194]^ and *vice versa*^[195]^. In fact, NF-κB was found to compete with Nrf-2 for the transcriptional co-activator, CREB binding protein^[195]^. It was also reported that the physical association of the N-terminal region of the p65 subunit of NF-κB with Keap1 can stabilize its association and inhibition of the Nrf-2 pathway^[196]^. This has significant implications on CRPC because NF-κB signaling is involved in PCa progression to androgen independence^[197]^. Furthermore, exposure to the Nrf2-activator, CDDO-me was shown to target HSPs, especially Hsp90^[198]^. Crosstalk between Nrf-2, NF-κB and AR signaling may contribute to the transition of PCa cells to CRPC cells by persistent AR signaling, as depicted in Figure 4.

In the following sections, we have focused our attention on how the activation of Nrf2 may be used as a highly effective approach to suppress the amplified redox signaling networks that enable the constant activation of NF-κB and AR signaling pathways in CRPC cells. We previously observed that Nrf-2 can decrease the transactivation function of AR^[150]^. Nrf-2 overexpression downregulated AR expression under basal conditions and decreased nuclear AR levels under DHT-stimulated conditions, and this effect was seen in both androgen-dependent (LNCaP) and androgen-independent (C4-2B) prostate cancer cell lines. Therefore, findings on several Nrf-2 activating agents are presented below.

## Potential of clinically-tested NRF2-activators in CRPC patients

Due to the complexity of redox regulated pathways, attempts to augment antioxidant signaling via simple removal of ROS with small molecule antioxidants will not be sufficient because a network of antioxidant proteins, transcription factors, and ROS producing enzymes work together to maintain the ideal oxidative environment within aggressive PCa cells. Due to the mortality and morbidity associated with CRPC progression, therapeutic strategies that target ROS-induced AR signaling and androgen-independence would be of significant importance. Hence, there is a crucial need to identify promising Nrf2-activating agents that may abrogate CRPCs.

Since the pathogenic manifestations of most chronic diseases share common mechanisms, including oxidative, inflammation and metabolic alterations such as ER-stress and insulin resistance, the targeting of Nrf2 and Keap1 presents significant and lucrative potential^[199,200]^. At least 30 recent patents for Nrf2 modulators (up- or down-regulators) are indexed in the World International Property Organization. Although several potent Nrf2-activators have been discovered in the past, most of these are still in an early stage of development. However, these Nrf2-activators represent a first attempt to structure Nrf2 as a common therapeutic and systems medicine approach. In the following section, we will discuss a few of the most promising Nrf2 activating agents that are currently under different phases of clinical trials^[200-204]^. These include: (1) approved and repurposed agents, such as Dimethyl fumarate (DMF) and Oltipraz; (2) compounds isolated from natural sources, like curcumin, sulforaphane and resveratrol; and (3) highly potent triterpenoid derivatives, e.g., RTA 402, RTA-408 and CXA-10 [Table 2].

**Table 2:** The Nrf2 activators currently in multiple clinical trials

NRF2 activator	Disease	Clinical trial
CDDO-Me (RTA-402)	Alport syndrome	Phase III
	Chronic kidney disease	Phase III
	Diabetic nephropathy	Phase III
	Liver disease	Phase II
	Pulmonary hypertension	Phase III
CDDO-DFPA (RTA-408)	Mitochondrial myopathy	Phase II
	Melanoma	Phase II
	Breast cancer	Phase II
Curcumin	Type 2 Diabetes mellitus	Phase IV
	Chronic kidney disease	Phase III
	Neoplasm	Phase II
	Prostate cancer	Phase III
CXA-10	Acute kidney injury	Phase I
Dimethyl fumarate (DMF)	Multiple sclerosis	Approved
	Psoriasis	Approved
	Rheumatoid arthritis	Phase II
	Cutaneous T cell lymphoma	Phase II
	Chronic lymphocytic leukemia small lymphocytic lymphoma	Phase I
Oltipraz	NASH	Phase III
Resveratrol	Type 2 diabetes mellitus	Phase I
	Endometriosis	Phase IV
	Colorectal cancer	Phase I
Sulforaphane	Prostate cancer	Phase II
	Lung cancer	Phase II
	Type 2 diabetes mellitus	Phase II
	Schizophrenia	Phase III

Although most of these compounds proved to be useful to some degree from a preclinical, proof-of-concept perspective, their clinical value to date has been generally very limited. Most of the potent Nrf2 activators are found to be electrophilic molecules that covalently modify, by oxidation or alkylation reactions, the cysteine residues present in the thiol-rich Keap1 protein. The majority of these Keap1 targeting agents are either repurposed agents, phytochemicals or pharmacological Nrf2 inducers.

### Repurposed agents

Two previously approved drugs, dimethyl fumarate (DMF) and Oltipraz, are now being repurposed as potent anti-inflammatory agents due to their ability to increase Nrf2 nuclear localization at clinically achievable concentrations [Table 2]. Fumaric acid esters are known to be the most prominent examples of Keap1 modifiers. DMF is approved by the FDA for the treatment of psoriasis and multiple sclerosis (MS). Although it is not a cure for MS, it helps decrease the number of episodes and worsening MS symptoms by suppressing the acute inflammatory phase and chronic autoimmune dysfunction^[205-208]^.

Interestingly, Linker *et al*.^[209]^, (2011) had shown that parallel activation of both Nrf2-dependent and Nrf2-independent mechanisms are associated with the therapeutic benefits of DMF. Indeed, DMF is a potent inhibitor of the NF-κB transcription factor^[210]^ which enables it to rapidly decrease proinflammatory cytokines, adhesion molecules^[211]^ and neutrophil infiltration^[212]^. Clinical studies have demonstrated that DMF reduces inflammatory cytokines, inhibits the maturation of dendritic cells, and increases apoptosis of dendritic cells and T cells. Promising pre-clinical and clinical studies have also shown that the antioxidant effects of DMF facilitates the prevention of endothelial dysfunction in diabetes, atherosclerosis, kidney dysfunction, and other cardiovascular complications^[213]^.

Preclinical studies in tumor models have also shown significant antitumor activity of DMF, which has been linked to its ability to activate Nrf2 and inhibit the NF-κB pathways^[214,215]^. Promising results have been observed with DMF in several types of cancer models such as glioblastoma, breast cancer, KRAS mutated cancers and myelogenous leukemia^[216-219]^. However, to our knowledge, the efficacy of DMF in prostate cancer patients, and especially, its ability to suppress CRPC progression has not been investigated so far. In light of our past findings on the suppressive effects of Nrf2 on AR expression in PCa cells^[150]^, and the *in vitro* efficacy of Nrf2-activators like sulforaphane^[70,220]^ and CDDO-me^[71]^ in similarly suppressing AR and increasing the efficacy of antiandrogens (discussed later in this section), we believe that DMF, a relatively safe and well tolerated Nrf2 activator, should be tested as a suppressor of CRPC outgrowth in PCa patients undergoing ADT treatment.

Oltipraz is another repurposed agent under clinical trials due to its Nrf2 activating ability. Oltipraz is an organosulfur compound that has been used as an anti-schistosomal agent for a number of years^[221]^ and this compound is now being repurposed as an anti-inflammatory agent for liver diseases^[222]^. It is thought to manifest its therapeutic effects by inhibiting the inflammatory and oxidative stress induced pathologic progression of liver fibrosis^[223]^. Therefore, oltipraz is currently in several Phase-III trials for the treatment of nonalcoholic steatohepatitis (NASH). Due to its xenobiotic effects on liver enzymes (Cyp450), the Nrf2-mediated antioxidant effects of Oltipraz has also shown significant liver accumulation and promise in the treatment of NASH. However, there has been little evidence that this approved drug may be effectively repositioned for cancer treatment, primarily due to the side-effects and drug-drug interaction issues associated with high dose oltipraz use^[224]^. Since both the pharmacokinetics and pharmacodynamics of this drug are well established, it may be beneficial to investigate whether oltipraz has any suppressive effect on AR and NF-κB function in PCa cells and whether it may suppress CRPC progression.

### Natural compounds

Because of their time-tested safety and efficacy, a huge number of natural product-derived antioxidant compounds, which are known to increase Nrf2, are currently in various stages of clinical development. The potent Nrf2 inducing and NF-κB inhibitory effects of phytochemicals like curcumin, sulforaphane and resveratrol are currently in several late-stage clinical trials for a variety of different inflammation and oxidative stress [Table 2].

Curcumin, the active component of *Curcuma longa* (the Indian spice turmeric), has shown significant promise as a potent antioxidant and is in several late-stage clinical trials. Curcumin’s antioxidant properties are being investigated in the treatment of obesity, metabolic syndrome, and prediabetes. *In vivo* studies have clearly shown that curcumin intake decreases inflammation and protects the liver by upregulating Nrf2^[225]^. Several drug-development studies have indicated that the oral intake of curcumin reduces serum triglycerides, IL-1β, IL-4, and VEGF and increases lipoprotein lipases that are helpful in breaking down fat^[226,227]^. With respect to cancer, the well-known anti-inflammatory and anti-cancer properties, and the multimodal actions of this phytochemical, prompted a number of laboratories to investigate the utility of curcumin as an adjunct to chemotherapy^[228]^. In addition to its potent effects in suppressing NF-κB activation, curcumin can also inhibit the PI3K/Akt pathway and induce ER-stress in cancer cells^[229,230]^. In colon cancer cells, curcumin has also been shown to trigger cell death by increasing stress-induced autophagy^[231]^. In lung cancer cells, curcumin exhibited synergistic effects when combined with docetaxel^[232]^. Studies have also documented the therapeutic benefit of curcumin against aggressive PCa and CRPC progression, both *in vitro* and *in vivo*^[233-235]^. Our past publication also showed the *in vitro* chemosensitizing effects of curcumin in PCa cells, especially when combined with another stress-inducing agent, the approved anti-HIV drug nelfinavir^[236]^. In CRPC cells, similar to our findings, several other groups have also documented that exposure to curcumin can suppress both AR expression and activity and increase the efficacy of antiandrogens. Interestingly, this effect was not only observed in prostate cancer cells^[233,237,238]^ but also in endocrine resistant (triple negative) breast cancer cells that express AR protein^[239]^. However, these therapeutic effects of curcumin were evident only at high concentrations (≥ 10 µmol/L), which is difficult to achieve *in vivo*^[234,240]^. Hence, numerous pharmaceutical companies are trying to develop more potent curcumin analogs, e.g., ASC-J9 or dimethylcurcumin^[241]^ and liposomal formulations of curcumin, to increase its bioavailability^[242]^. As early as 2012, Yamashita *et al*.^[243]^, (2012) had shown that ASC-J9 suppresses CRPC growth through the degradation of both full-length and splice variants of AR. However, a direct mechanistic link to the role of oxidative stress and the targeting of NF-κB or the Nrf2 pathways by ASC-J9 was not postulated in this manuscript. As documented above, the crosstalk between AR and NF-κB transcription factors is crucial for augmenting CRPC progression, where the suppressive effects of this Nrf2-activating phytochemical may be a promising candidate in abrogating endocrine resistance in PCa patients.

Another promising natural Nrf2 activating compound is sulforaphane, an isothiocyanate chemical present in cruciferous vegetables, such as broccoli^[244]^. Similar to curcumin, the mechanism primarily responsible for sulforaphane’s action was found to be potent suppression of both NF-κB DNA binding and transcriptional activation of the NF-κB regulated genes, apparently by modulating intracellular redox conditions^[245]^. Therefore, this natural compound is in several clinical trials to treat numerous inflammation-associated chronic diseases including cancer, asthma, chronic kidney disease, type 2 diabetes mellitus, and schizophrenia^[224,246,247]^. In type 2 diabetes mellitus, sulforaphane decreased gluconeogenesis and helped achieve glycemic control by increasing nuclear translocation of Nrf2^[248]^. Sulforaphane also reduced cardiovascular risk by decreasing serum triglycerides, and inflammatory markers including C-reactive protein and IL-6. Interestingly, studies have shown that SFN may also induce Nrf2 by decreasing TGF-β and JAK/STAT signaling^[249,250]^. In the kidney, sulforaphane protects renal cells by decreasing ROS and inhibiting both NF-κB and TGF-β signaling^[251]^.

Sulforaphane has been tested for both its chemopreventive^[252,253]^ and anticancer^[254]^ effects. Similar to curcumin, two of the most critical pathways by which sulforaphane displays its potent effect in PCa cells are Nrf2 activation and NF-κB inhibition. Furthermore, exposure to purified sulforaphane decreased proliferation in TRAMPC1 PCa cells *in vitro*^[255]^ and a diet rich in broccoli sprouts augmented Nrf2 expression and suppressed tumor development in the TRAMP (transgenic adenocarcinoma of mouse prostate) mouse model^[256]^. The reduction in tumor growth was reported to be linked with the stimulation of both Nrf2 and HO-1 proteins and inhibition of the Keap1 protein^[257]^. Furthermore, we recently showed that exposure to sulforaphane decreases AR levels and increases the efficacy of anti-androgens in PCa cells^[70]^. Most importantly, this hormone sensitizing efficacy of sulforaphane was also documented in the CRPC cell line, CWR22Rv1, further underscoring the therapeutic potential of Nrf2 activators.

A third phytochemical, which is produced by plants in response to injury or pathogens, is also showing promise as a safe Nrf2-activating agent^[258]^. Resveratrol is a stilbenoid, a type of natural phenol, and is found in high quantities in the skin of grapes, blueberries, raspberries, mulberries, and peanuts. Due to its favorable and safe pharmacokinetics profile, resveratrol is in several late-stage clinical trials^[259]^. Indeed, resveratrol prevents cardiovascular, inflammatory, and metabolic complications in hypertension, diabetes, heart disease, and atherosclerosis^[260]^. It decreases inflammatory markers including IL-1β and TNF-α by the downregulation of Keap1 expression, leading to the activation of Nrf2 signaling^[261,262]^. Resveratrol is also showing significant promise as an anti-cancer agent, against breast^[263]^, colorectal^[264]^ and prostate cancers^[265]^; however, most of these studies have been directed towards its chemopreventive effects and not as a therapeutic against endocrine resistance. Resveratrol was found to enhance the degradation of both AR and AR-V7 in prostate cancer cells^[266]^. Interestingly, in a recent study by De Amicis *et al*.^[267]^, (2019), resveratrol’s anticancer effects in both breast and prostate cancer cells was found to be the targeting of steroid hormone receptor signaling. Therefore, the above findings highlight the existing viability and significance of the use of natural products as sources of new candidate Nrf2-activator drugs to test in PCa patients undergoing hormone deprivation therapy.

### Triterpenoid derivatives

Synthetic triterpenoids are very potent Nrf2 activators and are effective even at nanomolar concentrations^[181,268]^. These are derivatives of 2-cyano3,12-dioxo-oleana-1,9(11)-dien-28-oate (CDDO; bardoxolone, RTA-401) that resemble the natural product oleanolic acid. In fact, the derivative CDDO-methyl ester (CDDO-Me, RTA-402) was first chosen to be in clinical trials for the treatment of diabetic nephropathy^[269]^. However, it had to be discontinued due to the risks associated with cardiovascular complications^[270]^. Currently, because of its potency and favorable *in vivo* pharmacokinetics^[271]^, CDDO-Me is currently under clinical investigation for potential treatment in Alport syndrome and pulmonary hypertension^[272]^. Indeed, CDDO-Me is one of the most well-studied synthetic triterpenoids showing potent anticarcinogenic activities^[273]^. Studies with CDDO-Me have been conducted in various kinds of cancers such as prostate^[274]^, breast^[275]^, ovary^[276]^, lung^[277]^, pancreatic^[278]^, and osteosarcoma^[279]^. Most importantly, unlike the phytochemical Nrf2 activators, CDDO-me has been shown to be effective at low nanomolar concentrations. At these physiologically achievable concentrations, CDDO-me was shown to reduce intracellular ROS levels via the transcriptional induction of numerous antioxidant proteins, e.g., SOD and Gpx, leading to a synchronized antioxidant and anti-inflammatory response^[280]^. *In vivo* studies have reported potent inhibitory effects of CDDO-Me on tumor growth, metastasis and angiogenesis and has demonstrated promising anticancer effects in Phase I clinical trials against advanced solid tumors and lymphomas^[281]^. However, although multiple studies with CDDO-Me have been conducted using PCa cells^[274,282,283]^, its efficacy to suppress CRPC progression has not been thoroughly investigated. In our recently published investigations, we documented that CDDO-Me suppresses both gene expression and protein levels of full-length AR and AR variants^[71]^. Furthermore, co-treatment with physiologically achievable doses of CDDO-Me was able to sensitize PCa cells to the cytotoxic effects of a clinically approved anti-androgenic drug, enzalutamide. These findings implicate the potential of CDDO-Me as an adjunct therapy to ADT in patients with CRPC tumors, especially those overexpressing AR and demonstrate a constitutive AR function. Due to the significant morbidity and mortality associated with CRPC progression and the crucial role of oxidative stress and NF-κB, it is imperative that this potent Nrf2-activating agent be initiated in a clinical trial to document its safety and efficacy in PCa patients undergoing hormone deprivation.

A number of laboratories are trying to synthesize safer and more potent triterpenoid derivatives with possible clinical applications as anti-inflammatory agents due to their Nrf2 activating potential. Another CDDO derivative, CDDO-difluoropropionamide (RTA-408, Omaveloxolone) with a better safety profile, is currently under clinical trials for myopathy^[284]^. Indeed, this newly synthesized triterpenoid has been shown to have a broad anticancer and anti-inflammatory activity^[285]^. Due to its effects in suppressing oxidative stress, omaveloxolone has been used in Friedreich’s ataxia models to suppress mitochondrial defects^[286]^ and to increase the regenerative capacity of diabetic wounds^[287]^. By suppressing chronic inflammation and immune dysfunction, this Nrf2 activator has also been shown to attenuate osteoclast production^[288]^ and nonalcoholic steatohepatitis (NASH)^[289]^. A third oleic acid derivative currently under early stage clinical trial is 10-nitro oleic acid (NO2-OA) or CXA-10^[290]^. Because of its enhanced bioavailability, this new Nrf2-activating compound is in trials for the potential treatment of acute kidney injury^[291]^. In light of the crucial role of inflammatory mechanisms that are activated in PCa cells undergoing hormone deprivation, therapeutic strategies that may suppress the vicious cycle of redox signaling that enable the selection and outgrowth of CRPC tumors need to be developed soon. The above mentioned Nrf2 activators, which are at different stages of clinical trials and have been shown to be relatively safe under chronic treatment settings, may have profound therapeutic value as an adjunct therapy in patients undergoing ADT.

There are several important caveats that need to be addressed towards the possible use of potent antioxidants such as Nrf2 activators towards the suppression of CRPC outgrowth post-ADT. It should be noted that Nrf2 plays a dual role in both cancer initiation and progression, and in dictating the efficacy of anticancer agents. During the early stages, Nrf2 has a protective role with respect to cancer development by inactivating the ROS-induced mutagenic effects of carcinogens. Indeed, the loss of Nrf2 function can promote neoplastic transformation of primary cells^[149,152]^. However, once tumors have developed, the activation of Nrf2 may protect tumor cells from ROS-mediated cytotoxicity, which is essential for the success of both radiotherapy and chemotherapy^[292]^. On the contrary, the acquisition of hormone resistance in PCa cells may be suppressed by exploiting the potent antioxidant effects of Nrf2-activating agents. Indeed, Nrf2 expression is often downregulated in CRPC cells^[15,64]^. Thus, chemo/radio-resistance and ADT resistance may be due to the opposing effects of Nrf2, which underscore the crucial importance of careful evaluation during the clinical application of Nrf2-activators in patients with early stage PCa. Furthermore, since a gradual adaptation to oxidative stress occurs during the acquisition of resistance to both chemotherapy and radiotherapy, the utility of Nrf2-activators in suppressing the development of endocrine resistance may need to be understood properly prior to their use as an adjunct to ADT. In this respect, a recent study by Ciamporcero *et al*.^[293]^ (2018) showed that crosstalk between Nrf2 and YAP transcription factors help maintain the antioxidant capacity of bladder cancer cells, which facilitate their acquisition of chemoresistance. Therefore, the therapeutic potential Nrf2 activation strategies to suppress CRPC outgrowth post-ADT will need to be directed towards the blockade of novel AR-independent mitogenic signaling networks in PCa cells.

For decades, different strategies to suppress the crucial AR-mediated mitogenic signaling in PCa cells have been the treatment of choice in ADT^[33,38,46]^. In addition to suppressing the sources of androgens, combination therapy with antiandrogens has been used to suppress AR signaling more fully. In recent years, alternate strategies to suppress AR protein levels in PCa cells by using inhibitors of AR chaperones (Hsp70, Hsp90), which increases AR degradation via the proteasomes, have shown some promise^[294,295]^. Several bromodomain inhibitors that prevent protein-protein interactions between the BET proteins, acetylated histones, and the AR transcription factor, are also being tested for CRPC patients^[296,297]^. Attempts to suppress the crosstalk between AR and IL-6 signaling using monoclonal antibodies against IL-6 or IL-6 receptors are also being investigated in order to decrease CRPC development^[105,130]^. With new evidence that CRPC cells may need a simultaneous suppression of multiple redox regulated signaling mechanisms, the therapeutic potential of Nrf-2 activators in PCa cells undergoing hormone deprivation needs to be understood. Combination therapy with these AR suppressive mechanisms may be used as an adjunct to ADT to abrogate endocrine resistance in prostate cancer.

## Conclusion

**Figure 5:**
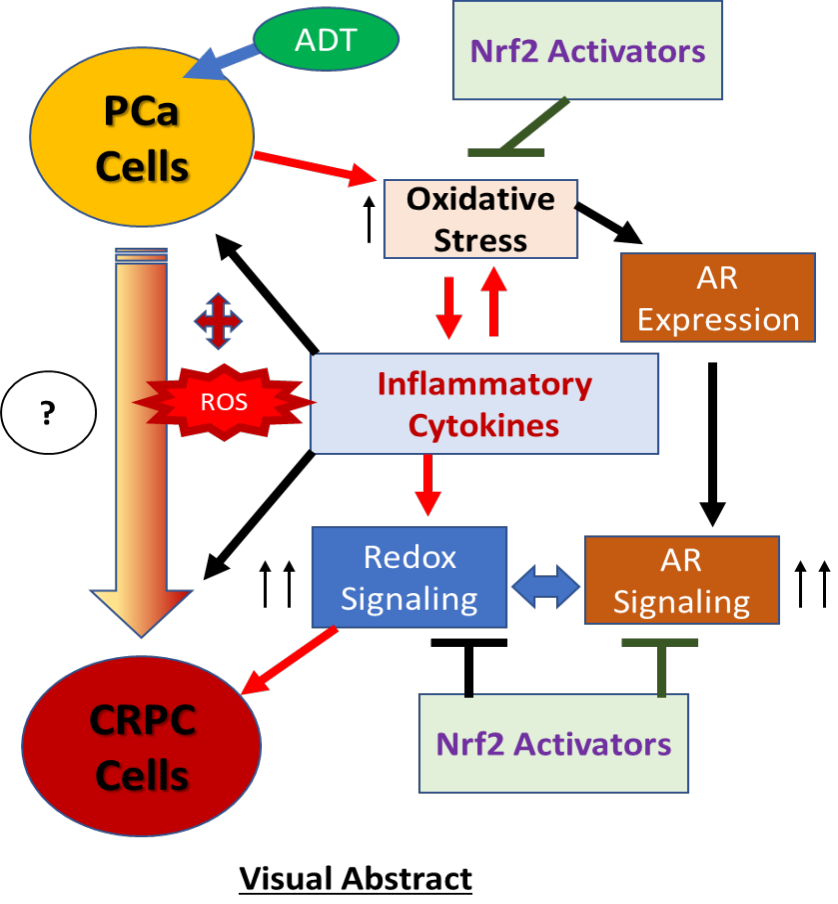
Therapeutic potential of Nrf2 activators in suppressing ADT-induced oxidative stress and CRPC progression. Androgen deprivation increases oxidative stress in PCa cells, which causes inflammation and redox signaling amplification in surviving tumor cells. This vicious cycle of signaling crosstalk between AR and non-AR signaling pathways facilitates the selection and outgrowth of CRPC. Potent Nrf2 activators, especially those under clinical trials, may abrogate this vicious cycle and suppress the development of endocrine resistant PCa. CRPC: castrate resistant prostate cancer; AR: androgen receptor; ADT: androgen deprivation therapy

Numerous past studies have suggested that hormone-deprivation induced oxidative stress and redox signaling in PCa cells can lead to the selection of cells with a CRPC phenotype. An inflammatory tumor microenvironment, which occurs due to the increased synthesis and secretion of multiple cytokines and chemokines by the tumor cells, and both tumor-associated stromal cells and macrophages, may amplify the second messenger signaling in the surviving PCa cells. Findings from numerous studies suggest that ADT-induced oxidative stress enables both the tumor cells and the tumor microenvironment to express inflammatory cytokines, which may further amplify the levels of ROS generation and enable a vicious cycle of signaling crosstalk with AR and non-AR signaling, resulting in the outgrowth of endocrine resistance. Therefore, mechanisms that regulate the pro- and anti-oxidant activities in PCa cells undergoing hormone deprivation, as well as therapeutic strategies that may be used to suppress this vicious cycle, may be an effective strategy to suppress CRPC progression [Figure 5]. In this respect, the regulation of redox signaling and antioxidant gene expression by the Nrf2 transcription factor may have multiple effects on abrogating the endocrine resistant phenotype of PCa. An integrated signaling network involving NF-κB and AR plays a critical role in persistent AR signaling and the development of CRPC, where potent Nrf2 activators, especially those under clinical trials for numerous other ailments, may be of significant therapeutic value [Figure 5].

## Declarations

### Copyright

© The Author(s) 2021.
